# Water Manganese Exposure and Children’s Intellectual Function in Araihazar, Bangladesh

**DOI:** 10.1289/ehp.8030

**Published:** 2005-08-09

**Authors:** Gail A. Wasserman, Xinhua Liu, Faruque Parvez, Habibul Ahsan, Diane Levy, Pam Factor-Litvak, Jennie Kline, Alexander van Geen, Vesna Slavkovich, Nancy J. LoIacono, Zhongqi Cheng, Yan Zheng, Joseph H. Graziano

**Affiliations:** 1 Department of Psychiatry, College of Physicians and Surgeons, Columbia University, New York, New York, USA; 2 New York State Psychiatric Institute, New York, New York, USA; 3 Department of Biostatistics,; 4 Department of Environmental Health Sciences, and; 5 Department of Epidemiology, Mailman School of Public Health, Columbia University, New York, New York, USA; 6 Gertrude H. Sergievsky Center, Columbia University, New York, New York, USA; 7 Lamont-Doherty Earth Observatory, Columbia University, Palisades, New York, USA; 8 Queens College, City University of New York, New York, New York, USA

**Keywords:** children, IQ, manganese

## Abstract

Exposure to manganese via inhalation has long been known to elicit neurotoxicity in adults, but little is known about possible consequences of exposure via drinking water. In this study, we report results of a cross-sectional investigation of intellectual function in 142 10-year-old children in Araihazar, Bangladesh, who had been consuming tube-well water with an average concentration of 793 μg Mn/L and 3 μg arsenic/L. Children and mothers came to our field clinic, where children received a medical examination in which weight, height, and head circumference were measured. Children’s intellectual function was assessed on tests drawn from the Wechsler Intelligence Scale for Children, version III, by summing weighted items across domains to create Verbal, Performance, and Full-Scale raw scores. Children provided urine specimens for measuring urinary As and creatinine and were asked to provide blood samples for measuring blood lead, As, Mn, and hemoglobin concentrations. After adjustment for sociodemographic covariates, water Mn was associated with reduced Full-Scale, Performance, and Verbal raw scores, in a dose–response fashion; the low level of As in water had no effect. In the United States, roughly 6% of domestic household wells have Mn concentrations that exceed 300 μg Mn/L, the current U.S. Environmental Protection Agency lifetime health advisory level. We conclude that in both Bangladesh and the United States, some children are at risk for Mn-induced neurotoxicity.

Manganese, a transition metal, is an essential nutrient in humans and animals. Like many other essential metals, excessive exposure has been associated with adverse health effects, in this case, neurotoxicity. Mn deficiency is rare in humans because the element is ubiquitous in common foods. Typically, dietary Mn intake greatly exceeds that from drinking water; exposure from water is usually small. Nevertheless, the World Health Organization (WHO) has established a health-based water Mn (WMn) standard of 500 μg/L (WHO 2002). Similarly, the U.S. Environmental Protection Agency (EPA) recently issued a drinking water health advisory for Mn that yielded a lifetime health advisory value of 300 μg/L in drinking water (U.S. EPA 2004).

Although the evidence linking inhalation exposure to neurotoxicity is compelling, evidence that links Mn exposure from drinking water to adverse neurologic effects is unconvincing. Occupational inhalation exposures in adults (Gorell et al. 1999; Mena et al. 1967; Takeda 2003) have repeatedly been associated with neuromotor consequences—specifically, akinetic-rigid Parkinsonism—largely due to toxic effects on dopaminergic neurons of the basal ganglia. Very little research has examined the consequences of excessive Mn exposure on neurologic or developmental functioning in children.

Last year, from a cross-sectional study of 201 10-year-old children in Bangladesh, we concluded that water arsenic (WAs) exposure was adversely associated with intellectual function (Wasserman et al. 2004). In the same study, we observed a moderate and statistically significant positive association between WAs and WMn (Spearman *r* = 0.39, *p* < 0.0001). Roughly 75% of the wells in our study region contained As in excess of the maximum contaminant level of 10 μg/L (van Geen et al. 2003), and 80% had levels of Mn that exceeded the WHO standard of 500 μg/L (Cheng et al. 2004). Among those 201 children, well WMn was adversely associated with children’s intellectual functioning; however, the magnitude of the association was reduced by approximately half (and was no longer statistically significant) when we adjusted for WAs. It was thus unclear whether or not WMn had an independent association with intellectual functioning. To facilitate interpretation, we sought an additional sample of children with low WAs.

Thus, to test the hypothesis that Mn might have an independent adverse effect on cognitive function, we returned to our Bangladesh study region. Our original As study included 54 children using wells with low As concentrations, that is, ≤ 10 μg/L. In our subsequent fieldwork, we recruited 88 additional children drinking from wells comparably low in As. Here we report on associations between WMn and intellectual function in the combined sample of 142 children.

## Materials and Methods

### Overview

Our present project is part of a larger ongoing multidisciplinary study by health, earth, and social scientists working collaboratively in Araihazar, Bangladesh. The study was approved by the Bangladesh Medical Research Council and the Columbia University Medical Center institutional review boards. We have previously described the region and the larger cohort study of adults, whose children are the focus of this investigation (Wasserman et al. 2004). As in most of rural Bangladesh, people in Araihazar live in houses with floors made from mud or cement, with roofs and walls constructed from concrete, tin, or straw. Members of extended families live in clusters of individual houses (a *bari*), surrounded by family farmland. Each *bari* has one or more tube wells, usually owned by a senior family member. This region is not particularly poor by Bangladeshi standards. Before conducting this study, we secured review and approval from institutional review boards at Columbia and in Bangladesh and obtained written informed consent from parents as well as child assent.

### Subjects

Details regarding the enrollment of the original 54 children are available in a previously published study (Wasserman et al. 2004). Briefly, in 2002, of the 11,749 adults enrolled in our cohort study, we selected 400 of their children at random (using 400 different wells) between 9.5 and 10.5 years of age; we ultimately assessed 201 children, 54 of whom were drinking from a well with WAs concentration ≤ 10 μg/L and who are included in these analyses. In 2004, we identified an additional 407 children who met three inclusion criteria: *a*) Their well had ≤ 10 μg As/L, *b*) their estimated age from earlier parental interviews was 9.5–10.5 years, and *c*) neither they nor their siblings had participated in our previous study. We limited the sample to children who were currently attending school. Of the first 199 families visited between May and November 2004, 88 met the inclusion criteria and consented to participate. Of the remaining 108 children, 76 were not of the desired age, 8 did not attend school, 11 had relocated, 12 refused to participate, and 1 was physically disabled.

### Procedure

Children and their mothers came to our field clinic, where the children participated in assessments described below and received a medical examination by a study physician. Weight, height, and head circumference were measured. In addition, children provided spot urine specimens for the measurement of urinary As (UAs) and urinary creatinine (UCr) and were asked to provide a blood sample for the measurement of blood Mn (BMn), blood As (BAs), blood lead (BPb), and hemoglobin (Hgb) concentrations. Of the 142 children assessed, 95 agreed to provide blood samples. Because our original intent was to measure only BPb and BAs, Mn-free needles were not used. The anticoagulant had negligible concentrations of Mn, Pb, and As. Blood and urine samples were frozen at –20°C and transported on dry ice to New York. Information on family demographics (e.g., parental education, occupation, housing type) was obtained from the baseline interview of parents during their enrollment in the cohort study. Information on the primary source of drinking water was obtained from the child’s mother. Parents were asked their age, education, and occupation; whether their home included a television; and the birth order of their children. As an additional surrogate for social class, the type of roofing on the well-owner’s home was recorded as thatched, tin, or cement and subsequently ranked on a scale (thatched, lowest; cement, highest). Children were given a toy as a sign of appreciation for their participation; all families participating in the larger cohort study continue to receive primary medical care at our own field clinic.

## Measures

### Water analyses.

Water samples were collected at the onset of the cohort study as part of a survey of all wells in the study region. Field sample collection and laboratory analysis procedures are described elsewhere in detail (Cheng et al. 2004; van Geen et al. 2003, 2005). In brief, samples were collected in 60-mL acid-cleaned polyethylene bottles, and 1 mL 7 N high-purity HCl was added for preservation before being shipped to Columbia University’s Lamont-Doherty Earth Observatory for analysis. Initially, some samples were analyzed for As only by graphite-furnace atomic absorption spectrometry. For the present study, all samples were reanalyzed by high-resolution inductively coupled plasma mass spectrometry (HR ICP-MS). The analytical detection limit of the method is 0.1 μg/L; the standard deviation of a single measurement is conservatively estimated at 4 μg/L (van Geen et al. 2005). Mn concentrations were also determined by HR ICP-MS. The detection limit of the method is also 0.1 μg/L, and its precision was 2% (Cheng et al. 2004).

### Urinary measurements.

UAs concentrations were assayed by graphite-furnace atomic absorption at the Mailman School of Public Health at Columbia University, using a Perkin-Elmer Analyst 600 system (PerkinElmer, Shelton, CT) as described previously (Nixon et al. 1991). Our laboratory participates in a quality-control program coordinated by P. Weber at the Québec Toxicology Center (Sainte-Foy, Québec, Canada). During the course of this study, intraclass correlation coefficients between our laboratory’s values and samples calibrated at Weber’s laboratory were 0.99. UAs levels were also adjusted for UCr concentrations, which were analyzed by a colorimetric Sigma Diagnostics Kit (Sigma, St. Louis, MO). Blood Hgb levels were determined by standard methods.

### ICP-MS blood measurements.

Venous whole blood samples were analyzed for BPb, BMn, and BAs concentrations in the Trace Metal Core Laboratory at the Mailman School of Public Health, which used a Perkin-Elmer Elan DRC II ICP-MS equipped with an AS 93+ autosampler. ICP-MS-DRC methods for metals in whole blood were developed according to published procedures (Pruszkowski et al. 1998; Stroh 1988), with modifications for blood sample preparation as suggested by the Laboratory for ICP-MS Comparison Program (Institut National de Santé Publique du Québec). A 3-mL EDTA Vacutainer of whole blood was thawed, thoroughly mixed, and then diluted 50 times with the following diluent: 1% HNO_3_, 0.2% Triton-X-100, 0.5% NH_4_OH. The sample was then centrifuged for 10 min at 3,500 rpm, and the supernatant used for analysis. One multielement standard solution was used for instrument calibration. The metal concentrations of that solution were chosen to cover the expected ranges of analyte concentrations in the blood samples: 5, 25, and 50 μg/L. Special attention was given to correction for matrix-induced interferences. Matrix suppression is compensated very well by the selection of suitable internal standards (IS), which are matched to masses and, if possible, to ionization properties of the analytes. For As, we used iridium (Ir); for Pb and Mn, we used lutetium (Lu) and gallium (Ga), respectively. A stock IS spiking solution was prepared that ultimately delivered to each tube 10 ng of Lu and Ir, and 100 ng Ga. After calibrating the instrument, we ran quality control samples, that is, blood samples with known analyte concentrations obtained from the Laboratory for ICP-MS Comparison Program.

Quality-control blood samples were purchased to cover the range of concentrations of analytes of interest and were run during the course of each day. Over a period of 1 month, during which all of these study samples were analyzed, the intraprecision coefficients of variation for BPb, BMn, and BAs were 1.5, 4.0, and 4.3%, respectively. In late 2004, when these samples were run, we also joined the Québec Multi-Elements External Quality Assessment Scheme run by the Laboratory for ICP-MS Comparison Program. Three times per year, that lab sends blood urine and serum samples with known concentrations of 23 elements. Only one blood sample was received and analyzed during the course of this study, but our reported concentrations for BPb, BMn, and BAs were well within the expected target ranges.

### Children’s intellectual function.

The Wechsler Intelligence Scale for Children, version III (WISC-III) (Wechsler 1991), suitable for children ≥ 6 years of age, consists of five (or six, depending on administration) verbal subtests that together provide a Verbal IQ score, and a similar number of performance subtests that together provide a Performance IQ. Neither the WISC-III (Wechsler 1991) nor any other recently well-standardized child IQ test has been adapted or standardized for use in Bangladesh.

In Araihazar, living conditions differ dramatically from those in the Western settings where this test was developed, which necessitated adaptations of the test for use in this culture. For example, a typical Araihazar home consists of a single room, often with a dirt floor. Most families use biomass fuel (leaves, hay, dung) for cooking. Electricity is available in most homes, typically consisting of one or two bulbs used for lighting. Many common Western household items, such as telephones and bathtubs, are rare.

We have previously described our adaptations of the WISC-III for this population (Wasserman et al. 2004). In short, we used six subtests that seemed the most culturally adaptable to this cultural context. Of the WISC-III Verbal subtests, we used Similarities and Digit Span; of the Performance subtests, we used Picture Completion, Coding, Block Design, and Mazes. As noted previously (Wasserman et al. 2004), we summed items across Verbal, Performance, and Full-Scale domains to create Verbal, Performance, and Full-Scale raw scores and also transformed these into measures of estimated Verbal, Performance, and Full-Scale IQ using procedures presented in the test manual (Wechsler 1991), despite the obvious limitations in application to this population.

Maternal intelligence was assessed with Raven’s Standard Progressive Matrices, a non-verbal test relatively free of cultural influences (Raven et al. 1983).

### Translation and Training

All tests and interviews were translated (and back-translated) between Bangla (Bengali) and English. As noted above, items deemed to be culturally inappropriate were altered or omitted. Materials were piloted to ensure maternal and child comprehension. Subsequently, two interviewers were trained by a competent tester (G.A.W.) and continued with supervised practice sessions for 2 weeks. All written test responses were rechecked when data were sent to Columbia University for entry.

### Statistical Analyses

#### Outcomes.

Because of concerns regarding the application of U.S. standardization of the WISC-III to Bangladeshi children, we first conducted analyses that predicted Verbal, Performance, and Full-Scale raw scores. Because the psychometric properties of IQ scores are more familiar to readers, we also applied the same analytical models to the prediction of estimated Verbal IQ, Performance IQ, and Full-Scale IQ.

#### Covariate adjustment and missing data.

We adjusted our models for the same set of covariates described in our previous As study (Wasserman et al. 2004): maternal education (categorized as none, 1–5 years, and 6–13 years) and intelligence, house type (thatched roof or poorer, corrugated tin, concrete construction), family ownership of a television, and child height and head circumference. For one girl without height data, we substituted the mean height for other participating girls.

#### Analytical model.

Analyses first estimated differences in the three measures of intellectual function, based only on the sociodemographic maternal factors, using linear regression models. We then estimated the incremental association of exposures (WMn, UAs, and WAs) singly and in combination, measured continuously. We repeated our analyses, categorizing children into groups based on quartiles of WMn to illustrate dose–response relationships. To examine further the dose–response relationship between WMn and intellectual function, we subsequently stratified children into four approximately equal-sized groups, based on well WMn. Because results based on quartiles of exposure were similar to those based on cut-points used in policy statements, we present data arrayed by the cut points, which correspond to various policy guidelines: Group 1 (reference), Mn < 200 μg/L (*n* = 38; 27%); group 2, 200 ≥ Mn < 500 μg/L (*n* = 45; 32%); group 3, 500 ≥ Mn < 1,000 μg/L (*n* = 31; 22%); group 4, Mn ≥ 1,000 μg/L (*n* = 28; 20%).

We next repeated these analyses for the subset of 95 children who provided blood samples for BMn, BPb, and BAs, measured continuously. The following variables were log-transformed to normalize their distributions: BPb, BAs, BMn, UAs, UCr, WMn, and WAs. For the most part, analyses are based on *n* = 142 children; however, those considering BPb, BAs, and BMn are based on *n* = 95 children.

## Results

### Sample Characteristics

Table 1 presents descriptive information for all demographic, water, and biochemical variables. Average child age was 10 years; approximately half of the sample were male; roughly one-third of children had regular access to a television, and > 70% lived in a house with a tin roof. On average, mothers and fathers reported 3.1 and 3.9 years of education, respectively. Children providing blood samples did not differ on any measure of exposure or intellectual function or on sociodemographic characteristics from those not providing blood samples (data not shown).

### Exposure Characteristics

The mean WMn concentration was 795 μg/L, with a very wide range, from 4 to 3,908 μg/L. By design, the range of WAs concentrations was narrow (0.1–10 μg/L), with a mean of 3.0 μg/L. Table 2 presents a matrix of Spearman correlation coefficients among water, urine, and blood metal measurements. Despite the restricted range of As exposure in the present sample, correlations among measurements of As in water, urine, and blood were all significantly positive. WMn was also positively correlated with WAs and BAs, but not so highly correlated as to preclude examination of their independent effects on child intelligence. WMn was not associated with BMn.

To obtain rough estimates of Mn intake from drinking water, we first calculated the mean WMn concentration for each quartile of WMn. The means for the four quartiles were 103, 440, 801, and 1,923 μg Mn/L. Based on a recent U.S. Institute of Medicine (IOM) report (IOM Food and Nutrition Board 2004), we estimated daily water intake for 10-year-old boys and girls to be 2.4 and 2.1 L/day, respectively. For the four quartiles, the product of WMn concentration times daily water intake yielded estimates of daily Mn consumption (from water only) of 0.25, 1.06, 1.92, and 4.37 mg/day for boys and 0.21, 0.93, 1.68, and 3.82 mg/day for girls.

### Relationship between Covariates and Intellectual Function

Linear regression analyses, predicting test raw scores from the sociodemographic features retained in the final “core” model, revealed better scores among children who *a*) had more educated mothers; *b*) lived in more adequate dwellings; *c*) had access to television; *d*) were taller; and *e*) had a larger head circumference (data not shown). Collectively, these factors explained 25.0, 24.1, and 17.7% of the variances in Full-Scale, Performance, and Verbal raw scores, respectively.

### Relationship between Well WMn and Intellectual Function

As Table 3 shows, before adjustment for sociodemographic factors, WMn was significantly associated with Full-Scale, Performance, and Verbal raw scores (*B*-values = –5.20, –4.43, and –0.80; *p*-values < 0.001, 0.001, and 0.02, respectively), explaining 10, 10, and 4%, respectively, of the variances in scores. After adjusting for sociodemographic factors, WMn concentration remained significantly and negatively associated with all three scores (*B*-values = –4.35, –3.76, and –0.63; *p*-values < 0.001, 0.001, and 0.05, respectively) and also explained incremental portions of the variances in scores (6.3, 6.9, and 2.3%, respectively). The addition of WMn to the core regression models produced negligible changes in the associations between core model variables and intellectual function raw scores. All results were similar when “IQ” outcomes were substituted for raw scores (data not shown).

### Controlling for As Exposure

The addition of WAs to these regression models failed to change the pattern of associations between intellectual function and sociodemographic variables, or between intellectual function and WMn. Not surprisingly, given that we sampled only children with low levels of WAs, WAs was not significantly associated with intellectual function. Similarly, associations between WMn and intellectual function scores were unchanged when we adjusted for both UAs and UCr. Neither UAs nor UCr was associated with children’s intellectual function (data not shown).

### Dose–Response Relationships between Well WMn and Intellectual Function

To examine the dose–response relationship between WMn and intellectual function, we subsequently stratified children into four approximately equal-sized groups, based on well WMn.

#### Before adjustment.

Unadjusted for other contributors, children in group 1, compared with those in the other three groups with higher WMn, had higher Full-Scale scores: groups 2 and 4 were significantly different from group 1 (*B*-values = –11.93 and –23.80; *p*-values < 0.05 and 0.0001, respectively), whereas the finding for group 3 was in the same direction but did not achieve significance (*B* = –8.92, *p* = 0.09). Similarly, compared with children in group 1, children in groups 2, 3, and 4 had lower Performance scores (*B*-values = –10.42, –7.97, and –20.39; *p*-values < 0.05, 0.07, and 0.0001, respectively). Finally, compared with children in group 1, those in group 4 also had significantly poorer Verbal scores (*B* = –3.76, *p* < 0.005); children in groups 2 and 3 had poorer Verbal scores than did those in group 1, but not significantly so.

#### After adjustment.

**Figure 1:**
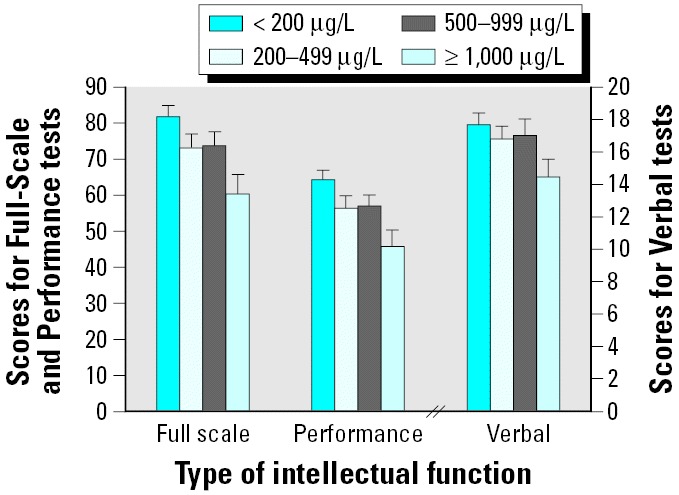
Adjusted and unadjusted scores by four groups of WMn for Full-Scale, Performance, and Verbal raw scores. In each case, adjustments were made for maternal education and intelligence, type of housing, child height and head circumference, and access to TV. Error bars indicate SEM.

Figure 1 illustrates the adjusted Full-Scale, Performance, and Verbal raw scores by WMn group. After adjustment for other factors, children in groups 1 and 4 were significantly different for Full-Scale, Performance, and Verbal scores (*B*-values = –21.28, –18.43, and –3.19; *p*-values < 0.0001, 0.0001, and 0.02, respectively). Compared with group 1, children in groups 2 and 3 had lower, albeit not significantly so, Full-Scale (*B*-values = –8.57 and –7.90, respectively; *p*-values < 0.10) and Performance scores (*B*-values = –7.79 and –7.34, respectively; *p*-values ≤ 0.07). Verbal score comparisons between children in groups 2 and 3 and those in group 1 were in the expected direction but did not approach significance.

#### Relations considering BPb, BAs, BMn, and intellectual function.

For the 95 children with blood samples, we examined the relations of BPb, BAs, and BMn to intellectual function, again adjusting for the same demographic features. When all three blood measures were added to the core model, only BPb was related to intellectual function (data not shown). In subsequent analyses that simultaneously considered WMn, WAs, and BPb, the adverse associations between WMn and Full-Scale and Performance scores persisted after adjustments (*B*-values = –4.56 and –3.82; *p*-values < 0.01).

## Discussion

This study indicates that exposure to Mn in drinking water is associated with neurotoxic effects in children. In our previous cross-sectional study of 10-year-old children in Araihazar, which reported an adverse association between WAs and child intellectual function, the mean WAs concentration was 118 μg/L, and the mean WMn concentration was 1,386 μg/L. In that study, before adjustment for WAs, WMn was adversely associated with children’s intellectual function, but the association did not persist once WAs was added to the regression model. The present study, however, was specifically designed to examine possible effects of WMn in the absence of confounding by WAs. WAs was controlled by limiting the sample to children drinking from wells with As ≤ 10 μg/L (with a mean of 3 μg/L), whereas WMn was free to vary. The lower mean Mn concentration of well water compared with the previous study (793 vs. 1,386 μg/L) reflects the fact that a significant fraction of the wells selected on the basis of their low As content tap older and deeper Bangladesh aquifers that are generally lower in Mn. This is the case not only in our study area but throughout the country (British Geological Survey and Department of Public Health and Engineering 2001; Cheng et al., 2004). Although the concentrations of the two elements were correlated (Spearman *r* = 0.36), exposure to As, as measured by water, urine, and blood concentrations, was essentially negligible, and none of the measures of As exposure was associated with any measure of intellectual function. In neither study have we detected a significant interaction between As and Mn exposure in relation to intellectual function; however, we lack adequate statistical power to definitively address this possibility.

The neurotoxicity of Mn in adults with occupational inhalation exposure is well established (Agency for Toxic Substances and Disease Registry 2001; Cook et al. 1974; Roels et al. 1999). The syndrome known as “manganism” is characterized by a Parkinson-like condition with weakness, anorexia, apathy, slowed speech, emotionless facial expression, and slow movement of the limbs. In contrast, findings from studies of environmental exposures to Mn are limited (Hudnell 1999; Mergler 1999; Mergler and Baldwin 1997). An epidemiologic investigation in Greece examined possible correlations between long-term (> 10 years) Mn exposure from drinking water and neurologic effects in a random sample of an elderly population (Kondakis et al. 1985); WMn concentrations ranged from 4 to 2,300 μg/L. Composite neurologic scores (including weakness/fatigue, gait disturbances, tremors) differed significantly between highly exposed and control populations. Two available studies of environmental Mn exposure in children have focused on motor functioning (He et al. 1994, as cited in Mergler 1999; Takser et al. 2003).

Among Parisian children followed from birth through their preschool years (Takser et al. 2003), after adjustment for sex and maternal education, cord BMn levels were negatively associated with scores on three scales derived by the authors from the McCarthy Scales (McCarthy 1972): attention, nonverbal memory, and hand skill. Among children 11–13 years of age, a comparison (unadjusted) of those from an area with high levels of Mn sewage irrigation with those from a control area revealed lower scores on tests of short-term memory, manual dexterity, and visuoperceptive speed in exposed children (He et al. 1994, as cited in Mergler 1999).

Mn is an essential element that is required by enzymes such as Mn superoxide dismutase and pyruvate carboxylase and serves to activate certain kinases, transferases, and other enzymes (WHO 2002). Substantial amounts are obtained in the diet, and deficiency is extremely rare. The first reported case of deficiency occurred in a man fed a chemically defined diet (as part of a study of vitamin K requirements) in which Mn was inadvertently left out (Doisy 1973). The IOM has determined the total adequate intake values for Mn for boys and girls, 9–13 years of age (the age group of interest to the present study) to be 1.9 and 1.6 mg/day, respectively (IOM Food and Nutrition Board 2002). One would therefore expect the shape of the dose–response relationship between WMn and cognitive function to be complex and dependent on dietary intake. We made no attempt to estimate dietary intake in this study of 10-year-old children and therefore cannot comment as to whether the WHO and U.S. EPA drinking water standards of 500 μg/L and 300 μg/L, respectively, are protective of the health of children.

Based on a comprehensive review of the literature, a risk assessment carried out by the IOM (IOM Food and Nutrition Board 2002) has generated age-dependent estimates of the tolerable upper intake level (UL), which is defined as the highest daily total dose of Mn that is likely to pose no risk of adverse health effects in almost all people. The UL for children 9–13 years of age was estimated to be 6 mg/day. In the present study, our estimates of water-borne Mn intake (by quartile of WMn) were 0.25, 1.06, 1.92, and 4.27 mg/day for boys, and 0.21, 0.93, 1.68, and 3.82 mg/day for girls. Obviously, none of these values exceed the UL of 6 mg/day, although additional dietary exposure could have pushed the total daily dose above that value. However, both the valence state and the bioavailability of Mn in food (oxidized Mn) and water (reduced Mn) differ, and these factors may contribute to the observed neurotoxicity of Mn from drinking water. The bioavailability of dietary Mn is very low, with estimates ranging from roughly 1 to 5% (IOM Food and Nutrition Board 2002); absorption is increased in the presence of iron deficiency (Finley 1999) and impaired by calcium supplementation (Freeland-Graves and Lin 1991). In contrast, it has been estimated that compared with that for food, the bioavailability of Mn from drinking water is 1.4 times greater in nonfasted subjects, and two times greater in fasted subjects (Ruoff 1995, cited in U.S. EPA 1999). Thus, dose for dose, water-borne Mn is likely more toxic than dietary Mn.

We found no evidence of a relationship between BMn and any measure of child intellectual function. This is not surprising because the use of blood levels as a means of evaluating occupational exposure to Mn has also been disappointing (National Research Council 1973). Our failure to use Mn-free needles may have introduced noise into the measurement of BMn and may explain the absence of an observed association between BMn and child intellectual functioning. As mentioned above, others (Takser et al. 2003) have observed a relationship between cord BMn and McCarthy scores at age 3. Because Mn is transported in the blood on transferrin, we speculate that serum Mn levels might be a better biomarker of exposure. Indeed, several reports indicate that serum or plasma Mn concentrations vary with dietary Mn intake (Davis and Greger 1992; Freeland-Graves and Turnlund 1996).

### Limitations.

We cannot comfortably make a statement about IQ points lost in relationship to WMn exposure, because we cannot apply U.S. standardization norms to generate IQ scores in the present study population. As we have previously pointed out, the lack of measures of intelligence, standardized for use in Bangladesh, hampers our ability to draw inferences about IQ points lost at given levels of exposure. Although we have followed sound procedures for adapting a widely used instrument to this very different cultural setting, and although we have avoided, for the most part, drawing conclusions about IQ, the measures used here are not measures of IQ, and the absence of standardized measures remains a limitation.

Our use of raw scores avoids pitfalls that would result from using nonstandardized procedures, but the removal of culturally bound items and subscales diverges from common practice. Nevertheless, the fact that other predictors of child intellectual function, such as maternal education and child height and head circumference, were significantly related to intellectual raw scores in the expected directions gives us confidence in the validity of the observed associations with Mn.

To date, we have studied only 10-year-old children, and we do not know if the observed deficits can be detected earlier in life. It is interesting to note that although breast milk contains between 3 and 10 μg Mn/L (Agency for Toxic Substances and Disease Registry 2001), infant formulas (Collipp et al. 1983) have been reported to contain as much as 50 to 300 μg/L. Our findings, coupled with the absence of reports of Mn deficiency in young children, led us to conclude that the possible consequences in children of excess exposure to Mn from water, diet, and gasoline additives (Kaiser 2003) deserve further attention.

We did not measure Mn in food or in air and thus could not estimate total Mn exposure. On the other hand, the impact of the absence of these exposure inputs would actually bias our findings toward the null. The fact that we observe a relationship between WMn and child intellectual function in the absence of estimates of food and air Mn exposure is therefore even more compelling. Moreover, because our original intent was to measure only BPb and BAs, Mn-free needles were not used.

### Implications.

**Figure 2:**
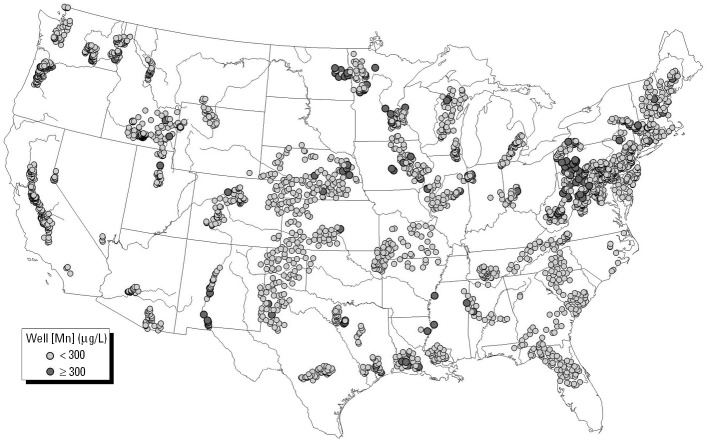
Distribution of the domestic household wells tested for Mn concentration based on data obtained by NAWQA of the USGS and downloaded at USGS (2005).

Our study findings led us to ask whether Mn exposure from drinking water might be a concern in the United States. Since 1991, the National Water-Quality Assessment Program (NAWQA) of the U.S. Geological Survey (USGS 2005) has systematically assessed the quality of source water for > 60% of the nation’s drinking water. Study areas were selected to represent a variety of important hydrologic and ecologic resources; critical sources of contaminants from agricultural, urban, and natural sources; and a high percentage of population served by municipal water supply and irrigated agriculture. Figure 2 and Table 4 illustrate the distribution of domestic household wells with Mn concentration > 300 μg/L obtained by the USGS NAWQA program. Based on the results of the present report, and the USGS finding that roughly 6% of domestic wells contain more than 300 μg/L, we believe that some U.S. children may be at risk for Mn-induced neurotoxicity.

## Correction

Some of the values were incorrect in the section “Dose–Response Relationships between Well WMn and Intellectual Function” and in Table 1 in the original manuscript published online; they have been corrected here.

## Figures and Tables

**Table 1:** Characteristics of study participants.^a^

Variable	Mean ± SD
Male^b^	70 (49.3)
TV access^b^	52 (36.6)
House type^b^	
Thatched roof or poorer	20 (14.1)
Corrugated tin	101 (71.1)
Concrete construction	21 (14.8)
Father’s occupation^b^	
Other/missing	19 (13.4)
Laborer/farmer	24 (16.9)
Factory/other paid job	49 (34.5)
Business	50 (35.2)
Child age	10.0 ± 0.4
Full-Scale IQ	64.5 ± 11.6
Verbal IQ	70.8 ± 12.2
Performance IQ	63.9 ± 11.9
Full-Scale raw score	71.2 ± 22.9
Verbal raw score	15.9 ± 5.4
Performance raw score	55.4 ± 18.9
Height (cm)	126.5 ± 6.7
Weight (kg)	22.4 ± 3.7
Body mass index	13.9 ± 1.3
Head circumference (cm)	49.5 ± 1.5
Mother’s education (years)	3.1 ± 3.5
Father’s education (years)	3.9 ± 3.8
Mother’s age (years)	33.8 ± 6.3
Mother’s Raven score	14.1 ± 3.1
WMn (μg/L)	795 ± 755
WAs (μg/L)	3.0 ± 2.6
UAs (μg/L)	57.5 ± 67.6
UCr (mg/dL)	45.4 ± 30.2
UAs (μg/g creatinine)	133.0 ± 86.8
Hgb^c^ (g/dL)	12.6 ± 1.1
BPb^c^ (μg/dL)	12.0 ± 3.7
BMn^c^ (μg/L)	12.8 ± 3.2
BAs^c^ (μg/L)	4.3 ± 1.9

a Except where noted, sample size is 142.

b Values reflect *n* (%).

c *n* = 95.

**Table 2:** Unadjusted associations (Spearman correlation coefficients) among measures of exposure to As, Pb, and Mn.

	UAs (μg/g creatinine)	WMn (μg/L)	BAs (μg/L)	BMn (μg/L)	BPb (μg/dL)
WAs	0.27**	0.36#	0.30**	–0.05	–0.06
UAs (μg/g creatinine)		0.16	0.51#	–0.03	–0.06
WMn			0.23*	–0.04	–0.13
BAs				0.02	–0.11
BMn					0.13

For comparisons between water and urinary concentrations, *n* = 142. Correlations involving whole blood metal concentrations are for the subset of 95 children who gave blood samples.

* *p* < 0.05,

** *p* < 0.01,

# *p* < 0.001.

**Table 3:** Predicting Verbal, Performance, and Full-Scale raw scores from WMn before and after covariate adjustment: unstandardized regression *B*-coefficient.

Variable	Full-Scale	Performance	Verbal
Before adjustment			
WMn (μg/L)	–5.20#	–4.43#	–0.80*
After adjustment			
Maternal education (years)			
None	–6.09	–2.95	–2.72*
1–5	–1.26	–0.37	–0.59
5–13^a^	—	—	—
Maternal intelligence	0.43	0.38	0.07
House type			
Thatched roof or poorer	–5.73	–6.02	0.21
Corrugated tin	–0.60	0.63	0.06
Concrete^a^			
TV access	2.32	1.98	0.48
Height (cm)	0.79**	0.62**	0.16*
Head circumference (cm)	3.48**	3.01**	0.50
WMn (μg/L)	–4.35#	–3.76#	–0.63*
Total *R*^2^ (%)	31.29	31.01	20.11

*R*^2^, total variance explained.

a Reference group.

* *p* < 0.05,

** *p* < 0.01,

# *p* < 0.001.

**Table 4:** Mn in U.S. domestic groundwater wells (n = 2,624).

Mn (μg/dL)	Frequency	Percent	Cumulative percent
< 200	2,386	90.9	90.9
201–300	81	3.1	94.0
301–500	71	2.7	96.7
501–1,000	56	2.1	98.9
> 1,000	30	1.1	100.0
